# Editorial for the Special Issue on Biosensors and MEMS-Based Diagnostic Applications

**DOI:** 10.3390/mi12030229

**Published:** 2021-02-25

**Authors:** Zeynep Altintas

**Affiliations:** Institute of Chemistry, Technical University of Berlin, Straße des 17. Juni 124, 10623 Berlin, Germany; zeynep.altintas@tu-berlin.de

Biosensors and micro-electromechanical systems (MEMS) have witnessed rapid development and enormous interest over the past decades. Constant advancements in diagnostic, medical, and chemical applications have been demonstrated with regard to several platforms and tools. Biosensors, relying on various sensing platforms such as surface plasmon resonance, piezoelectric, electrochemical, lab-on-a-chip, and paper, have been broadly used in research. Covering various excitation and readout schemes, MEMS devices transduce physical parameter changes, such as mass, temperature, or stress changes, caused by alterations in anticipated measurands, to electrical signals that can be further processed. Common examples of MEMS platforms include accelerometers, magnetic field sensors, pressure sensors, radiation sensors, microphones, and particulate matter sensors.

In total, nine papers are published in this Special Issue, covering biosensors and MEMS-based diagnostics applications [1,2,3,4,5,6,7,8,9]. In particular, Dou et al. fabricated a wearable contact lens sensor for non-invasive continuous monitoring of intraocular pressure (IOP) [1]. IOP is an important indicator of the diagnosis and treatment of glaucoma, as it has an apparent physiological rhythm and it generally reaches its peak value at night. To avoid missing the peak value at night and in order to sample the entire rhythm cycle, the continuous monitoring of IOP is essential. The authors engineered a wearable contact lens IOP sensor based on a platinum strain gauge using the micro-electromechanical process. The structure and parameters of the strain gauge were optimized to improve the sensitivity and temperature stability. Tests on an eyeball model indicated that the IOP sensor has a high sensitivity and significant dynamic cycling performance at different speeds of IOP variation. The non-invasive IOP sensor proposed in this report exhibits high sensitivity and satisfactory stability, with promising potential in continuous IOP monitoring [1]. Henriksson et al. proposed an approach to ring resonator biosensing assisted by dielectrophoresis, where they designed microring resonators with integrated electrodes next to the sensor surface, which may be used to explore the effect of dielectrophoresis [2]. The chip design was presented, which included two different electrode configurations, electric field gradient simulations, and the fabrication process flow for a dielectrohoresis-enhanced microring resonator-based sensor. Finite element method simulations were calculated for both electrode configurations around the sensing areas. The obtained results were comparable to the electric field gradients previously reported for successful interactions with larger molecules, such as proteins and antibodies [2]. Sardesai and coworkers reported on the design and electrochemical characterization of a spiral electrochemical notification coupled electrode platform for biosensing applications [3]. Microbial fuel cells (MFCs) are biological fuel cells based on the oxidation of fuels by electrogenic bacteria to generate an electric current in electrochemical cells. Şen-Doğan et al. proposed a new approach for the enhancement of the start-up time for microliter-scale microbial fuel cells (µMFCs) via the surface modification of gold electrodes [4]. The research findings showed that µMFCs modified with thiol self-assembled monolayers (cysteamine and 11-MUA) resulted in more than a 50% reduction in start-up times due to better bacterial attachment on the anode surfaces [4]. Sakamoto et al. examined the characteristics of the propagation of conduction in width-controlled cardiomyocyte cell networks in order to understand the contribution of the geometrical arrangement of cardiomyocytes to their local fluctuation distribution [5]. They tracked a series of extracellular field potentials of linearly ordered human embryonic stem cell-derived cardiomyocytes and mouse primary cardiomyocytes with 100 kHz sampling intervals for multielectrode signal acquisitions and used an agarose microfabrication technology to localize the cardiomyocyte geometries in the ordered cell networks with 100–300 μm wide agarose microstructures. This study pointed out that the “faster firing regulation” is not sufficient to explain the conservation of the propagation time distribution in cardiomyocyte networks, but rather should be expanded with a kind of cell network community effect, such as the lower fluctuation regulation [5]. Zhang et al. fabricated a bare optical-fiber-based biosensor to measure the refractive index values of different liquids and the binding kinetics of biomolecules to the sensor surface [6]. This optical fiber sensor was based on the Kretschmann’s configuration in order to attain the total internal reflection for surface plasmon resonance excitation. In the future, this device could serve as a useful biosensor for in situ measurement of allergens, antibody–antigen interactions, and circulating tumor cells in the blood [6].

This Special Issue also covers three comprehensive reviews written by the research groups of Külah, Altintas, and Bui and Medintz, respectively [7,8,9]. This reviews focuses on dielectrophoresis as a cell manipulation technique in BIOMEMS [7], the applications of graphene quantum dots in biomedicine and micro-supercapacitors [8], and the DNA microsystems for biodiagnosis [9].

I would like to take this opportunity to thank all of the authors for submitting their papers to this Special Issue. I also want to thank all of the reviewers for dedicating their time and helping to improve the quality of the submitted papers.
